# Nicotine from cigarette smoking and diet and Parkinson disease: a review

**DOI:** 10.1186/s40035-017-0090-8

**Published:** 2017-07-02

**Authors:** Chaoran Ma, Yesong Liu, Samantha Neumann, Xiang Gao

**Affiliations:** 1 Department of Nutritional Sciences, The Pennsylvania State University, University Park, University Park, PA USA; 2 Department of Neurology, Kailuan General Hospital, Tangshan, China; 3 Eberly College of Science, The Pennsylvania State University, University Park, University Park, PA USA

**Keywords:** Nicotine, Smoking, Diet, Gene-environment interactions, Parkinson disease

## Abstract

Evidence from epidemiological studies suggest a relationship between cigarette smoking and low risk of Parkinson disease (PD). As a major component of tobacco smoke, nicotine has been proposed to be a substance for preventing against PD risk, with a key role in regulating striatal activity and behaviors mediated through the dopaminergic system. Animal studies also showed that nicotine could modulate dopamine transmission and reduce levodopa-induced dyskinesias. However, previous clinical trials yield controversial results regarding nicotine treatment. In this review, we updated epidemiological, preclinical and clinical data, and studies on nicotine from diet. We also reviewed interactions between genetic factors and cigarette smoking. As a small amount of nicotine can saturate a substantial portion of nicotine receptors in the brain, nicotine from other sources, such as diet, could be a promising therapeutic substance for protection against PD.

## Background

Parkinson disease (PD) is an age-related neurodegenerative disorder, with a prevalence of 1–2% among adults aged 55 years and older [1]. It is characterized by a progressive degeneration of dopaminergic neurons in the substantia nigra pars compacta that results in tremor, rigidity, bradykinesia, and possibly dementia [2]. The current evidence related to the pathogenesis of PD includes defective handling of proteins, mitochondrial dysfunction, oxidative stress, and inflammation [3–6]. There is no cure for the disease and only symptomatic relief is available. Especially in the early stages of the disease, dopamine replacement therapies provide effective control of the motor symptoms with L-dopa as the gold standard. However, chronic L-dopa use does not adequately manage the non-motor deficits and additionally induces a variety of motor and psychiatric side effects that limits its effectiveness [7, 8]. These shortcomings strengthen the importance of identifying alternate treatment strategies that delay or halt disease progression, or ideally restore function in PD. Previous reviews reported the association between nicotine and PD risk, but the sources were limited to cigarette smoking and medical use. We thus updated epidemiological, preclinical and clinical data, and studies on nicotine from other sources. We also reviewed interactions between genetic factors and cigarette smoking to broaden the understanding of the potential protective role of nicotine on PD.

### Tobacco and PD

Previous epidemiologic studies have consistently shown that cigarette smoking [9–12] and smokeless tobacco use [13, 14] are associated with a lower risk of PD. Environmental tobacco smoke exposure is also associated with a significantly lower PD risk among never active smokers [15, 16]. Population-based studies have shown that smoking was associated with approximately 40–50% reduced risk of developing PD. [10, 11] This inverse relationship between smoking and PD was dose-dependent: age-adjusted relative risks (RRs) of PD were 0.8, 0.6, 0.5, and 0.4, for 1-9, 10-24, 25-44, and 45+ pack-years, relative to never-smokers, as shown in a large prospective cohort study [9]. There is also a temporal relationship between cigarette smoking and PD risk [9, 17]. Individuals with more years of smoking, older age at quitting smoking, and fewer years since quitting smoking had lower PD risk. Researchers prospectively observed a significantly lower risk of PD for smoking as early as 15 to 24 years before symptom onset, but not for smoking 25+ years before onset (*n* = 143,325) [17].

For smokeless tobacco use, such as tobacco chewing and snus use, three epidemiology studies have investigated their association with PD. [13, 14] One case-control study with 196 cases, have shown that tobacco use, including tobacco chewing or snuff use, is inversely associated with risk of PD. [13] In a cohort study with 9 years of follow-up, men who were current users of smokeless tobacco at enrollment had a significant lower risk of PD mortality (age-adjusted RR = 0.22, 95% confidence interval (CI), 0.07 to 0.67) [14]. Another prospective cohort study with 307 PD cases also had a similar inverse association between snus use and PD in men [18]. Consistently, in a case-control study based on 154 PD cases from Washington State, environmental tobacco smoke exposure was associated with 64% lower risk of PD. [15] Among persons with passive smoking as the only tobacco smoke exposure, risk was inversely associated with years exposed [15]. One cohort study using parental smoking as the tobacco exposure, has also shown the dose-response inverse association with PD incidence [16].

Because smoke has always been shown as a cause of adverse health outcomes, the inverse association between smoking and the risk of PD was counterintuitive. Some researchers believed that this relation resulted from a true biological protective effect of cigarette smoking. However, some researchers proposed that the reverse association might result from bias. For example, the association between smoking and lower PD risk could be explained by a still-unknown third factor that increases the risk of PD and also causes an aversion to smoking behavior [19]. A recent study reported that patients with PD were able to quit smoking more easily than controls [19]. This study suggests that the ease of smoking cessation is an early manifestation of pre-motor PD related to the loss of nicotinic rewards. In this case, quitting smoking could be just a pre-clinical marker rather than a risk factor [20]. However, in a case-control study in France, with 247 cases and 676 controls, when smoking was defined as cigarette smoking 18 years before PD onset, the same inverse association was still present [21].

Further, one of disadvantages of case-control studies is incidence-prevalence bias. This type of bias could be due to higher smoking-related mortality among incident cases than among controls, leading to a lower proportion of smokers among prevalent PD cases than among controls. However, the results of prospective cohort studies are in agreement with the results of case-control studies, which could minimize this type of bias. In addition, in a prospective study in the Health Professional Follow-up Study with 288 incident PD cases, smoking was not associated with a higher relative risk of death among PD patients than among non-PD patients [22]. An alternative hypothesis that genetic polymorphisms that influence tolerance to tobacco smoke may also increase the risk of PD might account this inverse association. Twin studies are usually used to test this type of hypothesis as a gold standard. An inverse relationship between smoking and PD was observed among monozygotic twins, suggesting that genetic factors is unlikely to confound this relationship [23, 24].

### Smoking and prodromal PD

REM sleep behavior disorder (RBD) is a parasomnia characterized by symptoms of dream-enacting behaviors and a loss of muscle atonia throughout a REM period as confirmed by polysomnography [25, 26]. A large proportion of individuals (>75%) with RBD developed neurodegenerative synucleinopathy based on prospective cohort studies [27–31]. However, in a hospital-based case-control study, RBD cases were more likely to smoke (adjusted OR = 1.43, *p* = 0.028), although nonsmoking status has been consistently linked with risk of PD. [32] In addition, two recent community-based cross-sectional studies [33, 34] showed that smoking was not a significant risk factor for probable RBD. For example, in our previous study based on 12,784 Chinese participants of the Kailuan study, the adjusted ORs for probable RBD was 0.91(95% CI: 0.74-1.1) for current smoking and 1.17 (95% CI: 0.85–1.6) for past smoking, compared with non-smokers [33]. In this context, it is possible that some RBD have different pathogenesis.

Both population-based studies [35, 36] and those performed in at-risk populations [37–40] showed that impaired olfactory function was associated with higher PD risk, and could predate development of PD [35]. With regard to the association between smoking and olfactory function, previous studies (all cross-sectional design) generated mixed results: some [41, 42], but not all [43–47], reported that smokers were more likely to have olfactory dysfunction. It remains unclear whether this is due to a true biological effect of smoking on olfaction or a reverse causality – individuals with olfactory dysfunction may quit smoking.

Constipation and higher risk of developing PD was observed in six population-based studies [48–53]. A cross-sectional study with 516 functional constipation cases reported an association between smoking and higher likelihoods of several functional gastrointestinal symptoms, including functional constipation [54]. Researchers observed that smoking delayed gastric emptying of solids, rather than liquids, and nicotine was not responsible for the effect [55], while acute cigarette smoking in habitual smokers delayed mouth–cecum transit time, an effect most likely due to nicotine [56]. After use of transdermal nicotine application in nonsmokers, a dose-dependent, significant decrease of total colon transit time was observed, mainly due to an accelerated transit in colon sigmoideum and rectum [57]. Additionally, colonic transit time was significantly shorter in men than in women, and smoking males have prolonged colonic transit time compared with nonsmoking men, while a difference was not observed in women [58]. However, in another cross-sectional study with 148 functional constipation cases in Bangladesh, smoking was not associated with functional constipation [59].

Regarding another risk factor for prodromal PD – erectile dysfunction [53, 60], multiple human, animal, case series, cross-sectional, and cohort studies support the notion that cigarette smoking is a risk factor for erectile dysfunction. Further, a positive dose-response relation has also suggested that increased quantity and duration of smoking is associated with a higher risk of erectile dysfunction [61].

### Mechanism of nicotine and PD

Findings on the inverse association between cigarette smoking and PD, together with observations that smokeless tobacco users had a lower risk of PD [14], support the notion that certain tobacco components, possibly nicotine, could be a promising substance for preventing against PD risk or slowing PD progression [62].

The rationale for the candidate role of nicotine is on the basis of evidence demonstrating a close anatomical relationship between the nicotinic cholinergic and dopaminergic neurotransmitter systems in the striatum [63]. Nicotine and its receptors play a key role in regulating striatal activity and behaviors mediated through the dopaminergic system [64], by activation of nicotinic acetylcholine receptors (nAChRs) on dopaminergic terminals and modulating dopamine release [65–67]. In experimental neuroscience, nicotine has shown beneficial effects -- nicotine or its agonists protect against 1-methyl-4-phenyl-1,2,3,6-tetrahydropyridine (MPTP) induced striatal damage and improve motor function in animal models of PD. [62, 64, 68–73] Nicotine and its agonists could also reduce levodopa-induced dyskinesias (LIDs) and had been evaluated in rodents and monkeys. In Parkinsonian monkeys, administration of nicotine reduced LIDs by 50–60%, without development of tolerance [74–77]. With respect to hemiparkinsonian rats, which received levodopa injection, nicotine also reduced dyskinesia by more than 50% [78, 79]. In vivo studies, researchers found that nicotine significantly reduced plasma levodopa concentration, and also observed that nicotine inhibited levodopa transport by Caco-2 cell monolayers in a manner of α-methyl amino isobutyric acid-independence and 2-amino-norbornanecarboxylic acid-dependence in vitro studies [80]. These results yield insight that nicotine may inhibit the transport of levodopa by the system L-amino acid transporter.

Regarding mechanism of neuroprotection against PD, another hypothesis states that the elevation of brain Cytochrome P450 enzymes (CYPs) induced by nicotine may play a role [81]. According to this hypothesis, higher levels of CYP enzymes in the brain, whether due to genotype or CYP induction by nicotine, can increase inactivation of neurotoxins, thus delaying development and progression of PD. [81] In addition, a novel hypothesis recently proposed that cigarettes may change the composition of the microbiota in the gut in a manner of mitigating intestinal inflammation [82]. Upregulation of anti-inflammation by nicotine would in turn lead to less misfolding of the protein alpha-synuclein, a substance inducing neurodegeneration in enteric nerves, thus minimizing propagation of the protein aggregates to the central nervous system [82].

### Dietary nicotine and PD

As demonstrated by a neuro-image study, a substantial portion of nicotine receptors became occupied when exposed to relatively small amount of nicotine [83]. This notion has further been supported by the observation that long-term smoking is more important than smoking intensity in the smoking-PD relationship [84, 85]. Besides cigarettes, nicotine has a wide distribution in flora and presents in some common vegetables that belong to the biological family of nightshades. They include potatoes, tomatoes, and peppers. Of note, the intake amount from these vegetables is generally much lower relative to that obtained from tobacco.

**Fig. 1:**
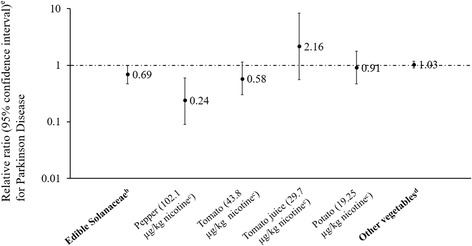
Parkinson disease risk associated with each additional serving of edible Solanaceaea and other vegetables [86]. ^a^ Among never-used-tobacco participants (241 PD cases and 239 controls) in a case-control study in western Washington State.^b^ All assessed edible Solanaceae: green, yellow or red peppers, tomatoes, tomato juice, and baked or mashed potatoes. ^e^ Relative risk (odds ratio) and 95% confidence interval, per once daily increase in typical adult life frequency of consumption, adjusted for age (continuous), sex, race/ethnicity, consumption of other vegetables (continuous) and caffeine (mg, continuous); and adjustment for or stratification by tobacco use (ever vs. never >100 cigarettes or regularly used cigars, pipes or smokeless tobacco). ^d^ All assessed non-Solanaceae vegetables combined: broccoli, cauliflower, cabbage, Brussels sprouts, radishes, lettuce, spinach/other greens, carrots, peas/lima beans, corn, sweet potatoes/yams, cucumbers, zucchini, yellow/winter squash and onions/scallions. ^c^ Median dry-weight nicotine concentration in each Solanaceae as typically consumed (e.g. ripe tomatoes and cooked potatoes) [116]

In a recent case-control study including 241 PD cases, each additional serving of edible solanaceae was associated with 31% lower risk of PD among never-smokers (Fig. 1) [86]. However, due to its retrospective design, possibility of reverse causality cannot be excluded, and some PD symptoms (e.g., olfactory dysfunction and difficulty swallowing) might change eating habits.

### Clinical trials

It is worth noting that unlike caffeine [87], urate [88–90] and flavonoids [91], which appear to be associated with lower risk of PD in only men but not in women, a strong inverse relationship between smoking and PD risk has been consistently observed in both men and women across populations. This relationship suggests that nicotine or other compounds in tobacco could be an excellent therapeutic agent.

Clinical trials and case studies regarding nicotine treatment have generated mixed results [62, 68, 92]. In addition to some benefits observed in case reports [93, 94], open-label studies yield mostly positive results [95–101], except for one of the eight open-label studies with no effect [102]. For those double-blinded placebo-controlled trials, three out of five reported no effect [103–105], while one showed worsening results [106] and one manifested positive results [107]. These studies are small (<80 PD cases) and such controversies may lie in discrepancies in the clinical stages of PD participants, administration of nicotine, and dosage and duration of nicotine treatment. Particularly, it is too late to do intervention by the time of clinical diagnosis of PD, which is at the late stage of the disease progression with irreversible impaired nervous system. Therefore, any intervention at this time might only have limited effects. In addition to those studies, recently three clinical trials are ongoing to the best of our knowledge. An open pilot study suggested that transdermal nicotine may improve motor scores and slow degeneration of dopaminergic neuron terminals [100]. The corresponding follow-up study with 40 PD patients (ClinicalTrials.gov ID NCT00873392) is complete, but published results are not presently available. Another study with 65 PD patients where an oral capsule of nicotine was given instead of a transdermal patch was completed, but to date no data have been published (NCT00957918). The third clinical trial is currently in progress to test whether transdermal nicotine can slow progression in early PD among 160 PD patients (http://clinicaltrials.gov identifier).

Further, some early small clinical trials reported poor tolerability of transdermal nicotine. For example, in a trial including 24 nonsmoking PD patients who received a transdermal nicotine treatment over 25 days, 14 participants had side effects such as nausea, vomiting, and dizziness, and 10 of them withdrew from the study [108]. These possible adverse effects highlight the importance of understanding the potential effect of low-dose nicotine obtained from other sources, such as edible solanaceae foods.

### Smoking-related gene and PD

Except positing that nicotine is neuroprotective, inherent aversion to nicotine-containing products could be another potential explanation. To date, several genetic studies have been conducted to explore the potential interaction between smoking and genetic factors on PD risk (Table 1). For example, a study including 677 PD patients reported a significant interaction between the nicotinic cholinergic receptor gene cluster CHRNA5 (rs588765) and smoking on age of onset of PD. [109] In an early study that included 466 singletons and 286 multiplex families, significant interactions between smoking and two SNPs (rs2255929 and rs1060826) of inducible nitric oxide synthase (iNOS) gene (NOS2A) in relation to PD risk were observed [110]. One possible interpretation for this observed interaction is that cigarette smoking condensates could attenuate inflammatory induction of iNOS, and reduce its cytotoxic effects [110].

**Table 1:** Gene-smoking interactions for Parkinson Disease

First author, year of publication	Study participants	Gene studied	Results
Greenbaum, 2013 [109]	667 Italian PD patients	CHRNA3, CHRNA4, and CHRNA5	A significant interaction between the CHRNA5 (rs588765) and smoking status (never smokers vs. ever-smokers)
Hancock, 2006 [110]	466 singleton and 286 multiplex families in the United States	NOS2A	Significant interactions of NOS2A (rs2255929 and rs1060826) with smoking in a subset of the families
Miyake, 2012 [113]	229 PD patients and 357 controls in Japan	SNCA	Significant interactions between SNCA (rs356219 and rs356220) and smoking with respect to sporadic PD
McCulloch, 2008 [112]	932 PD patients and 664 controls in the United States	MAPT, SNCA, UCHL1, and APOE	A significant interaction between SNCA REP1 and smoking
Gao, 2012 [91, 114]	584 PD patients and 1571 controls (non-Hispanic Caucasians)	SNCA, MAPT, SLC2A13, and HLA	A significant interaction with rs2896905 at SLC2A13
Palma, 2010 [115]	767 PD patients and 1989 controls in Europe	CYP1B1, CYP2D6, GSTM1, GSTM3, GSTT1, GSTP1, NQ01, SOD2, EPHX, NAT2, MAOA, MAOB, DAT1, and DRD2	Significant interactions between smoking and GSTM1 Pos/Null, GSTP1 haplotype, and NAT2 Fast/slow

Although earlier results were inconsistent, recent studies showed that a dinucleotide repeat polymorphism (REP1) in the promoter region of the α-synuclein gene (SNCA) was associated with the risk of common late-onset PD. [111] For example, a case-control study with 932 PD cases showed an interaction between SNCA REP1 and smoking on the risk of PD. [112] Another recent case-control study including 229 PD cases reported that smoking significantly modified the association between SNCA polymorphisms (rs356220 and rs356219) and PD risk [113]. In addition, many other genetic polymorphisms have been implicated in tobacco-PD relation, such as SLC2A13, GSTM1, NAT2, and GSTP1, etc., but the findings are inconsistent [114, 115].

## Conclusions

Despite of extensive evidence from epidemiological and basic research studies suggesting that nicotine may represent an effective agent with potential for prevention and alleviation PD, clinical data vary to a major extent between individuals. Discrepancies of those controversial results can be partly explained by differences of clinical stages of PD participants and research methodologies, and may also be due to the underlying gene-environment interactions. In addition, poor tolerability and high drop-out rate are inevitable concerns in such clinical trials. As a small amount of nicotine can saturate a substantial portion of nicotine receptors in the brain, nicotine from other sources, such as diet, could be a promising therapeutic substance for protection against PD.

## Abbreviations

CYPs: Cytochrome P450 enzymes
iNOS: Inducible nitric oxide synthase
LIDs: Levodopa-induced dyskinesias
MPTP: 1-methyl-4-phenyl-1,2,3,6-tetrahydropyridine
nAChRs: Nicotinic acetylcholine receptors
PD: Parkinson disease
REP1: Dinucleotide repeat
RRs: Relative risks
SNCA: α-synuclein gene

## References

[1] Bezard E, Brotchie JM, Gross CE (2001). Pathophysiology of levodopa-induced dyskinesia: potential for new therapies. Nat Rev Neurosci.

[2] Olanow CW, Tatton WG (1999). Etiology and pathogenesis of Parkinson’s disease. Annu Rev Neurosci.

[3] Bueler H (2010). Mitochondrial dynamics, cell death and the pathogenesis of Parkinson’s disease. Apoptosis.

[4] Zuo L, Motherwell MS (2013). The impact of reactive oxygen species and genetic mitochondrial mutations in Parkinson’s disease. Gene.

[5] Camilleri A, Vassallo N (2014). The centrality of mitochondria in the pathogenesis and treatment of Parkinson’s disease. CNS Neurosci Ther.

[6] Celardo I, Martins LM, Gandhi S (2014). Unravelling mitochondrial pathways to Parkinson’s disease. Br J Pharmacol.

[7] Connolly BS, Lang AE (2014). Pharmacological treatment of Parkinson disease: a review. JAMA.

[8] Huot P, Johnston TH, Koprich JB, Fox SH, Brotchie JM (2013). The pharmacology of L-DOPA-induced dyskinesia in Parkinson's disease. Pharmacol Rev.

[9] Hernan MA, Zhang SM, Rueda-deCastro AM, Colditz GA, Speizer FE, Ascherio A (2001). Cigarette smoking and the incidence of Parkinson's disease in two prospective studies. Ann Neurol.

[10] Hernan MA, Takkouche B, Caamano-Isorna F, Gestal-Otero JJ (2002). A meta-analysis of coffee drinking, cigarette smoking, and the risk of Parkinson's disease. Ann Neurol.

[11] Ritz B, Ascherio A, Checkoway H (2007). Pooled analysis of tobacco use and risk of Parkinson disease. Arch Neurol.

[12] Li X, Li W, Liu G, Shen X, Tang Y (2015). Association between cigarette smoking and Parkinson’s disease: A meta-analysis. Arch Gerontol Geriatr.

[13] Benedetti MD, Bower JH, Maraganore DM (2000). Smoking, alcohol, and coffee consumption preceding Parkinson's disease: a case-control study. Neurology.

[14] O'Reilly EJ, McCullough ML, Chao A (2005). Smokeless tobacco use and the risk of Parkinson’s disease mortality. Mov Disord.

[15] Searles Nielsen S, Gallagher LG, Lundin JI (2012). Environmental tobacco smoke and Parkinson’s disease. Mov Disord.

[16] O'Reilly ÉJ, Chen H, Gardener H, Gao X, Schwarzschild MA, Ascherio A (2009). Smoking and Parkinson’s disease: using parental smoking as a proxy to explore causality. Am J Epidemiol.

[17] Thacker EL, O'Reilly EJ, Weisskopf MG (2007). Temporal relationship between cigarette smoking and risk of Parkinson disease. Neurology.

[18] Liu Z, Roosaar A, Axell T, Ye W (2017). Tobacco use, oral health, and risk of Parkinson’s disease. Am J Epidemiol.

[19] Ritz B, Lee PC, Lassen CF, Arah OA (2014). Parkinson disease and smoking revisited: ease of quitting is an early sign of the disease. Neurology.

[20] Moccia M, Erro R, Picillo M (2015). Quitting smoking: an early non-motor feature of Parkinson’s disease?. Parkinsonism Relat Disord.

[21] Galanaud JP, Elbaz A, Clavel J (2005). Cigarette smoking and Parkinson’s disease: a case-control study in a population characterized by a high prevalence of pesticide exposure. Mov Disord.

[22] Chen H, Zhang SM, Schwarzschild MA, Hernan MA, Ascherio A (2006). Survival of Parkinson’s disease patients in a large prospective cohort of male health professionals. Mov Disord.

[23] Tanner CM, Goldman SM, Aston DA (2002). Smoking and Parkinson’s disease in twins. Neurology.

[24] Bharucha NE, Stokes L, Schoenberg BS (1986). A case-control study of twin pairs discordant for Parkinson’s disease: a search for environmental risk factors. Neurology.

[25] Frenette E (2010). REM sleep behavior disorder. Med ClinNorth Am.

[26] Iranzo A, Molinuevo JL, Santamaria J (2006). Rapid-eye-movement sleep behaviour disorder as an early marker for a neurodegenerative disorder: a descriptive study. Lancet Neurol.

[27] Postuma RB, Gagnon JF, Bertrand JA, Genier Marchand D, Montplaisir JY (2015). Parkinson risk in idiopathic REM sleep behavior disorder: preparing for neuroprotective trials. Neurology.

[28] Schenck CH, Boeve BF, Mahowald MW (2013). Delayed emergence of a parkinsonian disorder or dementia in 81% of older men initially diagnosed with idiopathic rapid eye movement sleep behavior disorder: a 16-year update on a previously reported series. Sleep Med.

[29] Iranzo A, Fernandez-Arcos A, Tolosa E (2014). Neurodegenerative disorder risk in idiopathic REM sleep behavior disorder: study in 174 patients. PLoS One.

[30] Wing YK, Li SX, Mok V (2012). Prospective outcome of rapid eye movement sleep behaviour disorder: psychiatric disorders as a potential early marker of Parkinson’s disease. J Neurol Neurosurg Psychiatry.

[31] Postuma RB, Iranzo A, Hogl B (2015). Risk factors for neurodegeneration in idiopathic rapid eye movement sleep behavior disorder: a multicenter study. Ann Neurol.

[32] Postuma RB, Montplaisir JY, Pelletier A (2012). Environmental risk factors for REM sleep behavior disorder: a multicenter case-control study. Neurology.

[33] Wong JC, Li J, Pavlova M (2016). Risk factors for probable REM sleep behavior disorder: a community-based study. Neurology.

[34] Ma JF, Qiao Y, Gao X (2017). A community-based study of risk factors for probable rapid eye movement sleep behavior disorder. Sleep Med.

[35] Ross GW, Petrovitch H, Abbott RD (2008). Association of olfactory dysfunction with risk for future Parkinson’s disease. Ann Neurol.

[36] Berg D, Marek K, Ross GW, Poewe W (2012). Defining at-risk populations for Parkinson’s disease: lessons from ongoing studies. Mov Disord.

[37] Postuma RB, Gagnon JF, Vendette M, Desjardins C, Montplaisir JY (2011). Olfaction and color vision identify impending neurodegeneration in rapid eye movement sleep behavior disorder. Ann Neurol.

[38] Ponsen MM, Stoffers D, Twisk JW, Wolters E, Berendse HW (2009). Hyposmia and executive dysfunction as predictors of future Parkinson’s disease: a prospective study. Mov Disord.

[39] Haehner A, Hummel T, Hummel C, Sommer U, Junghanns S, Reichmann H (2007). Olfactory loss may be a first sign of idiopathic Parkinson’s disease. Mov Disord.

[40] Mahlknecht P, Iranzo A, Hogl B (2015). Olfactory dysfunction predicts early transition to a Lewy body disease in idiopathic RBD. Neurology.

[41] Murphy C, Schubert CR, Cruickshanks KJ, Klein BE, Klein R, Nondahl DM (2002). Prevalence of olfactory impairment in older adults. JAMA.

[42] Vennemann MM, Hummel T, Berger K (2008). The association between smoking and smell and taste impairment in the general population. J Neurol.

[43] Le Floch JP, Le Lievre G, Labroue M, Paul M, Peynegre R, Perlemuter L (1993). Smell dysfunction and related factors in diabetic patients. Diabetes Care.

[44] Bramerson A, Johansson L, Ek L, Nordin S, Bende M (2004). Prevalence of olfactory dysfunction: the skovde population-based study. Laryngoscope.

[45] Liu G, Zong G, Doty RL, Sun Q (2016). Prevalence and risk factors of taste and smell impairment in a nationwide representative sample of the US population: a cross-sectional study. BMJ Open.

[46] Seubert J, Laukka EJ, Rizzuto D, et al. Prevalence and correlates of olfactory dysfunction in old age: a population-based study. J Gerontol A Biol Sci Med Sci. 2017. glx054. doi:10.1093/gerona/glx054. 10.1093/gerona/glx054 PMC5861894 28444135

[47] Huang Z, Huang S, Cong H (2017). Smell and taste dysfunction is associated with higher serum total cholesterol concentrations in Chinese adults. J Nutr.

[48] Abbott RD, Petrovitch H, White LR (2001). Frequency of bowel movements and the future risk of Parkinson’s disease. Neurology.

[49] Savica R, Carlin JM, Grossardt BR (2009). Medical records documentation of constipation preceding Parkinson disease: a case-control study. Neurology.

[50] Gao X, Chen H, Schwarzschild MA, Ascherio A (2011). A prospective study of bowel movement frequency and risk of Parkinson's disease. Am J Epidemiol.

[51] Lin CH, Lin JW, Liu YC, Chang CH, Wu RM (2014). Risk of Parkinson's disease following severe constipation: a nationwide population-based cohort study. Parkinsonism Relat Disord.

[52] Plouvier AO, Hameleers RJ, van den Heuvel EA (2014). Prodromal symptoms and early detection of Parkinson's disease in general practice: a nested case-control study. Fam Pract.

[53] Schrag A, Horsfall L, Walters K, Noyce A, Petersen I (2015). Prediagnostic presentations of Parkinson's disease in primary care: a case-control study. Lancet Neurol.

[54] Lundstrom O, Manjer J, Ohlsson B (2016). Smoking is associated with several functional gastrointestinal symptoms. Scand J Gastroenterol.

[55] Miller G, Palmer KR, Smith B, Ferrington C, Merrick MV (1989). Smoking delays gastric emptying of solids. Gut.

[56] Scott AM, Kellow JE, Eckersley GM, Nolan JM, Jones MP (1992). Cigarette smoking and nicotine delay postprandial mouth-cecum transit time. Dig Dis Sci.

[57] Rausch T, Beglinger C, Alam N, Gyr K, Meier R (1998). Effect of transdermal application of nicotine on colonic transit in healthy nonsmoking volunteers. Neurogastroenterol Motil.

[58] Meier R, Beglinger C, Dederding JP (1995). Influence of age, gender, hormonal status and smoking habits on colonic transit time. Neurogastroenterol Motil.

[59] Perveen I, Rahman MM, Saha M, Parvin R, Chowdhury M (2015). Functional constipation - prevalence and life style factors in a district of bangladesh. Mymensingh Med J.

[60] Gao X, Chen H, Schwarzschild MA (2007). Erectile function and risk of Parkinson’s disease. Am J Epidemiol.

[61] Biebel MG, Burnett AL, Sadeghi-Nejad H (2016). Male sexual function and smoking. Sex Med Rev.

[62] Quik M, Perez XA, Bordia T (2012). Nicotine as a potential neuroprotective agent for Parkinson’s disease. Mov Disord.

[63] Zhou FM, Wilson CJ, Dani JA (2002). Cholinergic interneuron characteristics and nicotinic properties in the striatum. J Neurobiol.

[64] Quik M, Huang LZ, Parameswaran N, Bordia T, Campos C, Perez XA (2009). Multiple roles for nicotine in Parkinson’s disease. Biochem Pharmacol.

[65] Grady SR, Salminen O, Laverty DC (2007). The subtypes of nicotinic acetylcholine receptors on dopaminergic terminals of mouse striatum. Biochem Pharmacol.

[66] Quik M, Wonnacott S (2011). alpha6beta2* and alpha4beta2* nicotinic acetylcholine receptors as drug targets for Parkinson’s disease. Pharmacol Rev.

[67] Quik M (2004). Smoking, nicotine and Parkinson’s disease. Trends Neurosci.

[68] Quik M, O'Leary K, Tanner CM (2008). Nicotine and Parkinson’s disease: implications for therapy. Mov Disord.

[69] Quik M, Bordia T, Huang L, Perez X (2011). Targeting nicotinic receptors for Parkinson’s disease therapy. CNS Neurol Disord Drug Targets.

[70] Janson AM, Fuxe K, Agnati LF, Sundström E, Goldstein M, Adlkofer F, Thurau K (1991). The effect of chronic nicotine treatment on 1-Methyl-4-Phenyl-1,2,3,6-tetrahydropyridine-induced degeneration of nigrostriatal dopamine neurons in the black mouse. Effects of Nicotine on Biological Systems.

[71] Janson AM, Fuxe K, Goldstein M (1992). Differential effects of acute and chronic nicotine treatment on MPTP-(1-methyl-4-phenyl-1,2,3,6-tetrahydropyridine) induced degeneration of nigrostriatal dopamine neurons in the black mouse. Clin Investig.

[72] Parain K, Marchand V, Dumery B, Hirsch E (2001). Nicotine, but not cotinine, partially protects dopaminergic neurons against MPTP-induced degeneration in mice. Brain Res.

[73] Bordia T, Parameswaran N, Fan H, Langston JW, McIntosh JM, Quik M (2006). Partial recovery of striatal nicotinic receptors in 1-methyl-4-phenyl-1,2,3,6-tetrahydropyridine (MPTP)-lesioned monkeys with chronic oral nicotine. J Pharmacol Exp Ther.

[74] Quik M, Mallela A, Chin M, McIntosh JM, Perez XA, Bordia T (2013). Nicotine-mediated improvement in L-dopa-induced dyskinesias in MPTP-lesioned monkeys is dependent on dopamine nerve terminal function. Neurobiol Dis.

[75] Quik M, Mallela A, Ly J, Zhang D (2013). Nicotine reduces established levodopa-induced dyskinesias in a monkey model of Parkinson’s disease. Mov Disord.

[76] Zhang D, Bordia T, McGregor M, McIntosh JM, Decker MW, Quik M (2014). ABT-089 and ABT-894 reduce levodopa-induced dyskinesias in a monkey model of Parkinson’s disease. Mov Disord.

[77] Zhang D, McGregor M, Decker MW, Quik M (2014). The alpha7 nicotinic receptor agonist ABT-107 decreases L-Dopa-induced dyskinesias in parkinsonian monkeys. J Pharmacol Exp Ther.

[78] Bordia T, Campos C, Huang L, Quik M (2008). Continuous and intermittent nicotine treatment reduces L-3,4-dihydroxyphenylalanine (L-DOPA)-induced dyskinesias in a rat model of Parkinson's disease. J Pharmacol Exp Ther.

[79] Bordia T, Campos C, McIntosh JM, Quik M (2010). Nicotinic receptor-mediated reduction in L-DOPA-induced dyskinesias may occur via desensitization. J Pharmacol Exp Ther.

[80] Kyaw WT, Nagai M, Kaneta M (2013). Effect of nicotine on the pharmacokinetics of levodopa. Clin Neuropharmacol.

[81] Miksys S, Tyndale RF. Nicotine induces brain CYP enzymes: relevance to Parkinson’s disease. J Neural Transm Suppl. 2006:177–80. 10.1007/978-3-211-45295-0_28 17017527

[82] Derkinderen P, Shannon KM, Brundin P (2014). Gut feelings about smoking and coffee in Parkinson’s disease. Mov Disord.

[83] Brody AL, Mandelkern MA, London ED (2006). Cigarette smoking saturates brain alpha 4 beta 2 nicotinic acetylcholine receptors. Arch Gen Psychiatry.

[84] Chen H, Huang X, Guo X (2010). Smoking duration, intensity, and risk of Parkinson disease. Neurology.

[85] Kenborg L, Lassen CF, Ritz B (2015). Lifestyle, family history, and risk of idiopathic Parkinson disease: a large Danish case-control study. Am J Epidemiol.

[86] Nielsen SS, Franklin GM, Longstreth WT, Swanson PD, Checkoway H (2013). Nicotine from edible Solanaceae and risk of Parkinson disease. Ann Neurol.

[87] Palacios N, Gao X, McCullough ML (2012). Caffeine and risk of Parkinson’s disease in a large cohort of men and women. Mov Disord.

[88] O'Reilly EJ, Gao X, Weisskopf MG (2010). Plasma urate and Parkinson’s disease in women. Am J Epidemiol.

[89] Gao X, Chen H, Choi HK, Curhan G, Schwarzschild MA, Ascherio A (2008). Diet, urate, and Parkinson’s disease risk in men. Am J Epidemiol.

[90] Gao X, O'Reilly EJ, Schwarzschild MA, Ascherio A (2016). Prospective study of plasma urate and risk of Parkinson disease in men and women. Neurology.

[91] Gao X, Cassidy A, Schwarzschild MA, Rimm EB, Ascherio A (2012). Habitual intake of dietary flavonoids and risk of Parkinson disease. Neurology.

[92] Thiriez C, Villafane G, Grapin F, Fenelon G, Remy P, Cesaro P (2011). Can nicotine be used medicinally in Parkinson’s disease?. Expert Rev Clin Pharmacol.

[93] Ling H, Petrovic I, Day BL, Lees AJ (2012). Smoking-induced transient motor deterioration in a levodopa-treated patient with Parkinson’s disease. J Neurol.

[94] Hanagasi HA, Lees A, Johnson JO, Singleton A, Emre M (2007). Smoking-responsive juvenile-onset Parkinsonism. Mov Disord.

[95] Moll H (1926). The treatment of post-encephalitig Parkinsonism by nicotine. Br Med J.

[96] Marshall J, Schnieden H (1966). Effect of adrenaline, noradrenaline, atropine, and nicotine on some types of human tremor. J Neurol Neurosurg Psychiatry.

[97] Ishikawa A, Miyatake T (1993). Effects of smoking in patients with early-onset Parkinson’s disease. J Neurol Sci.

[98] Trinh K, Andrews L, Krause J (2010). Decaffeinated coffee and nicotine-free tobacco provide neuroprotection in Drosophila models of Parkinson's disease through an NRF2-dependent mechanism. J Neurosci.

[99] Mitsuoka T, Kaseda Y, Yamashita H (2002). Effects of nicotine chewing gum on UPDRS score and P300 in early-onset parkinsonism. Hiroshima J Med Sci.

[100] Villafane G, Cesaro P, Rialland A (2007). Chronic high dose transdermal nicotine in Parkinson’s disease: an open trial. Eur J Neurol.

[101] Kelton MC, Kahn HJ, Conrath CL, Newhouse PA (2000). The effects of nicotine on Parkinson’s disease. Brain Cogn.

[102] Lemay S, Chouinard S, Blanchet P (2004). Lack of efficacy of a nicotine transdermal treatment on motor and cognitive deficits in Parkinson’s disease. Prog Neuro-Psychopharmacol Biol Psychiatry.

[103] Clemens P, Baron JA, Coffey D, Reeves A (1995). The short-term effect of nicotine chewing gum in patients with Parkinson’s disease. Psychopharmacology.

[104] Shoulson I (2006). Randomized placebo-controlled study of the nicotinic agonist SIB-1508Y in Parkinson disease. Neurology.

[105] Vieregge A, Sieberer M, Jacobs H, Hagenah JM, Vieregge P (2001). Transdermal nicotine in PD: a randomized, double-blind, placebo-controlled study. Neurology.

[106] Ebersbach G, Stock M, Muller J, Wenning G, Wissel J, Poewe W (1999). Worsening of motor performance in patients with Parkinson’s disease following transdermal nicotine administration. Mov Disord.

[107] Fagerstrom KO, Pomerleau O, Giordani B, Stelson F (1994). Nicotine may relieve symptoms of Parkinson’s disease. Psychopharmacology.

[108] Lemay S, Blanchet P, Chouinard S, Masson H, Soland V, Bedard MA (2003). Poor tolerability of a transdermal nicotine treatment in Parkinson’s disease. Clin Neuropharmacol.

[109] Greenbaum L, Rigbi A, Lipshtat N (2013). Association of nicotine dependence susceptibility gene, CHRNA5, with Parkinson’s disease age at onset: gene and smoking status interaction. Parkinsonism Relat Disord.

[110] Hancock DB, Martin ER, Fujiwara K (2006). NOS2A and the modulating effect of cigarette smoking in Parkinson’s disease. Ann Neurol.

[111] Maraganore DM, de Andrade M, Elbaz A (2006). Collaborative analysis of alpha-synuclein gene promoter variability and Parkinson disease. JAMA.

[112] McCulloch CC, Kay DM, Factor SA (2008). Exploring gene-environment interactions in Parkinson’s disease. Hum Genet.

[113] Miyake Y, Tanaka K, Fukushima W (2012). SNCA polymorphisms, smoking, and sporadic Parkinson’s disease in Japanese. Parkinsonism Relat Disord.

[114] Gao J, Nalls MA, Shi M (2012). An exploratory analysis on gene-environment interactions for Parkinson disease. Neurobiol Aging.

[115] De Palma G, Dick FD, Calzetti S (2010). A case-control study of Parkinson's disease and tobacco use: gene-tobacco interactions. Mov Disord.

[116] Siegmund B, Leitner E, Pfannhauser W (1999). Determination of the nicotine content of various edible nightshades (Solanaceae) and their products and estimation of the associated dietary nicotine intake. J Agric Food Chem.

